# A deep learning-based model for plant lesion segmentation, subtype identification, and survival probability estimation

**DOI:** 10.3389/fpls.2022.1095547

**Published:** 2022-12-15

**Authors:** Muhammad Shoaib, Babar Shah, Tariq Hussain, Akhtar Ali, Asad Ullah, Fayadh Alenezi, Tsanko Gechev, Farman Ali, Ikram Syed

**Affiliations:** ^1^ Department of Computer Science, CECOS University of IT and Emerging Sciences, Peshawar, Pakistan; ^2^ College of Technological Innovation, Zayed University, Dubai, United Arab Emirates; ^3^ High Performance Computing and Networking Institute, National Research Council (ICAR-CNR), Naples, Italy; ^4^ Department of Molecular Stress Physiology, Center of Plant Systems Biology and Biotechnology, Plovdiv, Bulgaria; ^5^ Department of Computer Science and Information Technology, Sarhad University of Science & Information Technology, Peshawar, Pakistan; ^6^ Department of Electrical Engineering, College of Engineering, Jouf University, Sakaka, Saudi Arabia; ^7^ Department of Plant Physiology and Molecular Biology, University of Plovdiv, Plovdiv, Bulgaria; ^8^ Department of Software, Sejong University, Seoul, Republic of Korea; ^9^ School of Computing, Gachon University, Seongnam-si, Republic of Korea

**Keywords:** plant lesion, disease detection, CANet CNN, classification and DICE coefficient, machine learning

## Abstract

Plants are the primary source of food for world’s population. Diseases in plants can cause yield loss, which can be mitigated by continual monitoring. Monitoring plant diseases manually is difficult and prone to errors. Using computer vision and artificial intelligence (AI) for the early identification of plant illnesses can prevent the negative consequences of diseases at the very beginning and overcome the limitations of continuous manual monitoring. The research focuses on the development of an automatic system capable of performing the segmentation of leaf lesions and the detection of disease without requiring human intervention. To get lesion region segmentation, we propose a context-aware 3D Convolutional Neural Network (CNN) model based on CANet architecture that considers the ambiguity of plant lesion placement in the plant leaf image subregions. A Deep CNN is employed to recognize the subtype of leaf lesion using the segmented lesion area. Finally, the plant’s survival is predicted using a hybrid method combining CNN and Linear Regression. To evaluate the efficacy and effectiveness of our proposed plant disease detection scheme and survival prediction, we utilized the Plant Village Benchmark Dataset, which is composed of several photos of plant leaves affected by a certain disease. Using the DICE and IoU matrices, the segmentation model performance for plant leaf lesion segmentation is evaluated. The proposed lesion segmentation model achieved an average accuracy of 92% with an IoU of 90%. In comparison, the lesion subtype recognition model achieves accuracies of 91.11%, 93.01 and 99.04 for pepper, potato and tomato plants. The higher accuracy of the proposed model indicates that it can be utilized for real-time disease detection in unmanned aerial vehicles and offline to offer crop health updates and reduce the risk of low yield.

## 1 Introduction

Crop development and yield are crucial factors that affect agriculture and farmers in every conceivable way, including economically, socially, and politically (Production et al., 2014). Consequently, monitoring the development of crops to detect various types of illness is a crucial step at specific times. However, naked human eye may not be sufficient, and occasionally deceptive scenarios may occur (Arsenovic et al., 2019). Automatic recognition and classification of diverse agricultural diseases are required for accurate identification. This paper provides an overview of the methods proposed for our research project. This paper contains the suggested methodology’s context, problem definition, objectives, and scope. Pakistani farmers’ illiteracy is one of the major contributors to a rise in microbial infections (Government of Pakistan, 2021). Once a disease has infected a crop, it is difficult for farmers to determine its root cause. Pathogens and pests are wreaking havoc on crops. This information comes from a study report published by UC Agriculture and Natural Resources; the crop increases the yield of five major food crops by 10 to 40% (Ali et al., 2017). In the context of Pakistan, where agriculture provides 16% of the GDP and employs over 60% of the people, it is essential to adopt extensive steps to prevent plant diseases. According to the Ministry of Food Processing Industries, agricultural losses in 2016 totaled thirteen billion US dollars (GoI, 2019). Image processing and neural networks can be used to perform one of the beneficial steps in plant disease diagnosis techniques (Tugrul et al., 2022). Recent research has demonstrated that neural networks and deep learning perform categorization tasks effectively.

Agriculture is a crucial sector in nations such as Pakistan, whose economies depend directly or indirectly on agriculture. It highlights the requirement of caring for plants from the seedling stage to the harvest. To produce the desired yield, the crop plants must endure sometimes unfavourable meteorological conditions, survive various diseases, and attacks by animals. The latter can be resolved if the crops are shielded from the various animals. Weather circumstances are beyond human control but there are technologies to mitigate abiotic stresses (Kerchev et al., 2020; Talaviya et al., 2020). Lastly, it is vital to safeguard the crop against various illnesses, as they can affect the crop’s total growth and output. If these diseases can be identified promptly, the crop can be safeguarded with the necessary agrochemicals. Digitalizing this disease diagnosis and classification procedure might be advantageous for farmers. It will reduce the time and accuracy required to identify and classify diseases.

Agriculture is the most ancient occupation that occurred even before science. With the advancement of science, it became clear that plants were living organisms capable of respiration, reproduction, and susceptible to certain diseases. These diseases, caused by many microorganisms, including bacteria, viruses, and fungi, diseases can cause significant damage to crops and can harm humans, as it is the case with pathogen-produced mycotoxins and other toxicants (Nan et al., 2022). Furthermore, destruction of crops by pathogens can cause human starvation. In 1840, a disease known as the Late blight destroyed a substantial potato crop. It is also known as the Irish famine, and it was a sad period in European history during which many people perished from hunger (Goss et al., 2014). As plants are vital to human survival, so we must safeguard them against deforestation and numerous plant diseases. A significant portion of Pakistan’s population is still engaged in agriculture. It ranks second in agricultural production worldwide. However, two major issues harm Pakistan’s crops: natural disasters and infectious diseases. According to United Nations data, agriculture lost 96 billion dollars in 2005-2015. We have no control over natural calamities, but we can control plant illnesses caused by microorganisms. In the context of Pakistan, where agriculture provides 16% of the GDP and employs over 60% of the people, it is essential to adopt extensive steps to prevent plant diseases. According to the Ministry of Food Processing Industries, agricultural losses in 2016 totaled thirteen billion dollars. Image processing and neural networks can be used to perform one of the beneficial steps in plant disease diagnosis techniques. Recent research has demonstrated that neural networks and deep learning perform categorization tasks effectively.

The proposed research work focuses on three plant species: potato, tomato, and pepper. In a poll, 61.33 percent of potato producers cited light as one of the primary causes of crop loss. In 2020, according to national statistics, almost 60 percent of tomato crops in Punjab failed to owe to a virus. Pakistan accounts for 40 percent of the world’s total pepper production. Additionally, pepper has numerous natural benefits for throat infections. China is the largest producer of tomatoes and potatoes, whereas India is the second-highest producer of these two crops. Enhancing fertilization and automating the disease detection system can increase agricultural yield in our country.

To our knowledge, plant leaf lesion segmentation, lesion subtype categorization, and overall survival prediction have been addressed individually without regard for their inherent linkages. This research uses deep neural networks along with some advanced machine learning techniques to provide a complete strategy for plant leaf lesion segmentation, the recognition of lesion subtypes, and the survival estimation of a plant. Detailed contributions are listed below. First, a novel context-aware Convolutional Neural Network (CNN)-based technique for plant lesion segmentation is presented. In the second phase, a hybrid model is utilized for plant survival estimation using the segmented ROI information. A context-encoded convolutional neural network (CANet) (Zhang et al., 2019) is employed to extract high-dimensional features which are classified by the linear regression machine learning methods to make plant life survival predictions. In the final phase of the proposed framework, all the distinct tasks, i.e., segmentation, classification, and survival estimation, are merged into a single interconnected deep learning strategy. In conclusion, whereas the plant damage tests and classification criteria recommend foliar and pathological images, the suggested method effectively detects plant disease using only leaf RGB image data. On the PlantVillage dataset, the proposed segmentation and classification scheme was validated.

The rest of this paper is organized as follows. Section 2 shows the literature review of Plant Lesion Segmentation using deep learning methods. Section 3 presents the methodology of the proposed models. Section 4 shows the experimental results. Finally, Section 5 concluded the proposed work.

## 2 Related work

Using a mix of a Deep Learning classification model (CNN) and a features selection method genetic algorithm (GA), a model is presented for the diagnosis and recognition of tomato plant disease using the leaf image data (Tugrul et al., 2022). The proposed given framework was trained on 500 images belonging to 4 types of diseases. The features learning block of the CNN model is used to extract important visual characteristics and for classification. In a research study, an examination of the efficacy of CNN architecture for the recognition of plant diseases using the leaf data was conducted to detect diseases in soybean plants (Yu et al., 2022), the framework is implemented using LeNet which is the smallest and simplest CNN architectures. The leaf photos of 13,842 images of soybean plants are gathered from the PlantVillage benchmark dataset. The above framework has an accuracy of 98.44%, demonstrating CNN’s usefulness for classifying plant diseases based on leaf images. The approach to plant disease identification involves the construction of a modern model for identifying 13 plant illnesses from photographs of healthy plant leaves (Sladojevic et al., 2016). Caffe, an architecture for deep learning, was used to train the data. The framework in question yielded outcomes with a 91 to 98 percent accuracy. The author of the research article (He et al., 2020) developed a two-stage approach. In the beginning, the architectures based on meta information of Regional Convolutional Neural Network (R-CNN), Regional Fully Connected Network (R-FCN), and Single Shot detector (SSD) are combined to develop a single object detector. The features learning blocks of VGG16 (Khattab, 2019), VGG-19 (Szymak et al., 2020), and inception-V3 (Szymak et al., 2020) are utilized to extract high-dimension features from the training data and evaluation of models performances. Comparing the proposed model to other similar detection models, the proposed model is found more time efficient. A novel deep-learning architecture is developed for the detection and recognition of mango plant diseases (Saleem et al., 2021). The proposed CNN is trained on 754 unhealthy and 780 healthy mango leaf image data. The custom framework achieves an average f1-score of 97.01%. The author (Durmus et al., 2017) suggested a system based on the architecture of convolutional neural networks to recognize and categorize several potato plant diseases. The dataset utilized for this framework contains 2,465 photos of potatoes. The author (Geetharamani and J., 2019) researched and recognized the benefits and cons of the model and the performance of deep learning neural networks, which are used to recognize and classify various plant diseases. The literature review and picture database experiments comprise 50,000 photographs of many plant diseases (Durmus et al., 2017). The author presented a novel deep learning-based framework that is capable of segmenting the affected region on the leaf and recognizing the type of disease in tomato plants (Islam et al., 2022). This framework’s dataset comprises 13,281 tomato leaf photos with nine types of illnesses which are collected from the PlantVillage dataset. The model achieved an average of 99.91% accuracy on the training data while on the testing data the average accuracy achieved is 98.96%. The author (Goss et al., 2014) concentrated on identifying and categorizing diverse diseases in rice plants using the CNN features and SVM classifier for decision-making. of a framework utilizing CNN architecture and SVM. The training data consists of 696 rice plant leaf images which belong to four types of rice plant diseases. Maximum accuracy of 91.37 percent is reached when evaluating the accuracies of diverse training and testing datasets. In the case of sugar beet, an existing model is upgraded, utilizing the faster region-based CNN architecture by modifying the parameters for recognizing disease-affected regions (Nasirahmadi et al., 2021). The dataset comprises 155 photos of sugar beets, and the proposed framework attained an accuracy rate of 95.48 percent. In the case of Olive plant diseases, the author of the research article (He et al., 2020) compared a transfer learning scenario with CNN architectures such as VGG-16 and VGG-19, as well as proposed CNN architectures (Alshammari et al., 2022). The framework applied to the dataset includes around 3,400 photos of Olive plant leaves. In this framework, a data augmentation technique was utilized to increase the size of the dataset. Before data augmentation, the accuracy was approximately 88%; after data augmentation, it was approximately 95%. The study paper (Abdulridha et al., 2020) proposes a CNN-based tailored model for tomato leaf disease detection. In addition, I compared the proposed model to models of machine learning and VGG-16. The proposed model achieved 98.4% accuracy, the KNN model achieved 94.9% accuracy, and the VGG-16 model achieved 93.5% accuracy. The dataset of tomato leaf pictures utilized by this framework is derived from the Plant village dataset. Deep learning’s transfer learning technique is used to detect and categorize illnesses using leaf images of two crops, such as cucumber and rice (Liu and Wang, 2021). The suggested framework was applied to 2,430 photos of cucumbers and rice afflicted with eight illnesses collected from the plant village dataset.

The proposed framework achieved a precision of 90.84 percent (Liu and Wang, 2021). The influence of deep learning on diagnosing plant illnesses using leaf photos was examined. CNN architecture functions as a black box model for plant disease diagnosis. Also covered are the many hyperparameter characteristics that affect classification accuracy. Numerous models and research have used deep learning scenarios to identify and classify illnesses in certain plant categories. Deep learning can also be used to identify and categorize the macronutrients present in a particular plant. The proposed technique for monitoring plant health checks several stages from the seedling stage through the yielding stage to increase yield. The suggested system was implemented using a dataset of 571 photos, including images of tomato leaves and tomato fruit at various stages of crop growth. The inception-ResNet v2 and autoencoder performance was 87.27 percent and 79.09 percent, respectively. This literature demonstrates the impact of transfer learning on identifying and categorizing plant diseases using photographs of leaf surfaces. According to the article’s author (Siddiqua et al., 2022), picture segmentation with the aid of colors, i.e., color image segmentation approaches, improves comprehension and problem-solving. One can first determine an image’s red, Green, and Blue color components. The red and green components aid in identifying the yellow portions of the image, typically indicated as infected. Fuzzy logic is an effective method for solving disease classification issues (Sibiya and Sumbwanyambe, 2021), the author proposes the minimum distance approach, a genetic algorithm modification, to locate a plant’s infected portion for picture segmentation (Ngugi et al., 2021). After picture segmentation, the author examined the accuracy of the technique using different classification algorithms, such as k mean clustering and SVM (Bargelloni et al., 2021). In this paper, the author uses a convolution neural network technique to diagnose various plant illnesses (Production et al., 2014; Saleem et al., 2021). The author has conducted an exhaustive study. Photographs of diverse plant leaves, including images of both sick and healthy leaves, are captured. The author has grouped it into numerous categories, and all CNN designs achieved an accuracy greater than 97%. AlexNet (Yoo et al., 2021), AlexNetOWTBn, GoogLeNet (Wang et al., 2015), Overfeat, and VGG are the CNN architectures. The author has comprehensively analyzed several deep learning algorithms, their benefits and drawbacks, and optimization strategies (Sarker, 2021). In the linked work, these strategies have also been compared. In this publication (Ngugi et al., 2021), the author describes the proposed algorithm in depth, the image acquisition was the initial step, followed by image enhancement and segmentation. The HSV approach was utilized for the segmentation of color images. Integrated into the instrument for evaluating plant disease were sensors that could determine the meteorological and climatic factors affecting the plant disease in real-time.

## 3 Methods

This section includes a comprehensive overview of the proposed DCNN model’s architecture and training method, including the preparation of the dataset and experimental procedures. The suggested model for detecting plant leaf diseases begins with dataset preparation and concludes with model prediction. Python 3.8, TensorFlow Library version 2.10.0, NumPy 1.23.4, matplotlib 3.6.1, and OpenCV 4.6.0 are used to prepare the training dataset and implement the proposed DCNN model, respectively. The simulations, i.e., model development, training, validation, etc., are performed on an HP Z440 workstation consisting of core i7 12 cores of CPU and a DDR4 ram of 48 GB. The proposed scheme also utilized NVidia RTX-3090 Graphical Processor Unit (GPU), which uses the CUDA framework to allow the parallel processing speeds up the proposed model training and testing procedure. The workstation for implementing the proposed DCNN is equipped with a dual Intel Xeon Silver 4310 (12 cores, 24 threads, and 2.10Ghz) processor and six Nvidia Tesla P100 GPUs to expedite the training of deep neural networks. The following sections will explain all the important phases of the proposed plant disease detection framework in detail. The section that follows addresses the specifics of data set preparation and preprocessing.

### 3.1 Setup and preprocessing of datasets

Images of damaged and Normal/Healthy plant leaves were retrieved from a typical open data collection (Geetharamani & J., 2019). Sixteen distinct plant species were used to compile a dataset on plant leaf diseases. Each plant comprises the dataset’s healthiest and most prevalent disease categories. There are 58 distinct plant leaves, with one category containing no specimens. Initial data collection yielded 61,459 plant leaf and leafless photos. **Table 1** displays the list of plant types and their corresponding classes consisting of the healthy and illness categories in the recommended benchmark dataset.

**Table 1:** PlantVillage Benchmark dataset Descriptions.

S. No	Plant Type	Disease Name
1	Pepper	Normal Bacterial Spot
2	Potato	Normal Early and Late Blight
3	Tomato	Normal Bacterial spot Early Blight Late Blight Leaf Mold Leaf Spot Spider Mite Target Spot Mosaic Virus Yellow Leaf Curl Virus

Each category now contains an even amount of photographs utilizing data augmentation techniques. By adding upgraded photos to the training dataset, data enhancement techniques can also expand the size of the dataset and prevent overfitting during model training. The images in the dataset are enhanced using the Generative adversarial network (GAN) and advanced image manipulation (AIM) and Neural style learning (NST) schemes that increase the quality of the image by automatically adjusting the contrast, removing noise, and sharpening the images. The AIM-enhancing techniques include image scaling, mirroring, Histogram based color improvement, and rotation. The histogram color enhancement method adjusts the strength of the three color channels R-G-B by adjusting the major pixel components (Tang, 2020).

In addition, picture cropping, tilting, shearing, and scaling make enhanced images through the modification of the input images’ hue, saturation, and location. A total of 36,541 images from the plant village dataset are enhanced using the AIM scheme. DCGAN generates an image enhancement that resembles the training image data. The DCGAN network is composed of a dual network i.e. a generator and a discriminator. The generator module of the network creates random noise and applies it to the training images.

In contrast, the discriminator section of the DCNN learns to identify real and synthetic pictures (Lu et al., 2019). The DCGAN network is trained with a training period of 10,000 and a short batch size of 64 on a graphics processing unit. The DCGAN enhancement technology added 32 million enhanced photos to the dataset. NST is an additional picture-generating method that employs deep learning algorithms. Using a modified VGG19 network, an NST enhancement model was built in this study. The NST model was trained on a deep learning server for 5,000 epochs. The NST model requires two distinct images as input and produces an enhanced image as output: the first image is a content image while the second image is a style reference image. The first image comprises the fundamental elements that should be integrated with the output image. The second image also known as the reference image applies a style pattern and integrates it into the final output image. NST image enhancement scheme adds up some common features from the style picture to the content image for generating the output image. In the dataset, the NST enhancement method produced 17,500 enhanced photos. Finally, AIM, NST, and GAN algorithms were utilized to improve the image and equalize the data counts for each dataset category. The name PlantVillage denotes the proposed dataset used in this research for proposed model validation. The number of photos in the dataset rose from 61,459 to 147,500 due to these enhancements.

**Figure 1:**
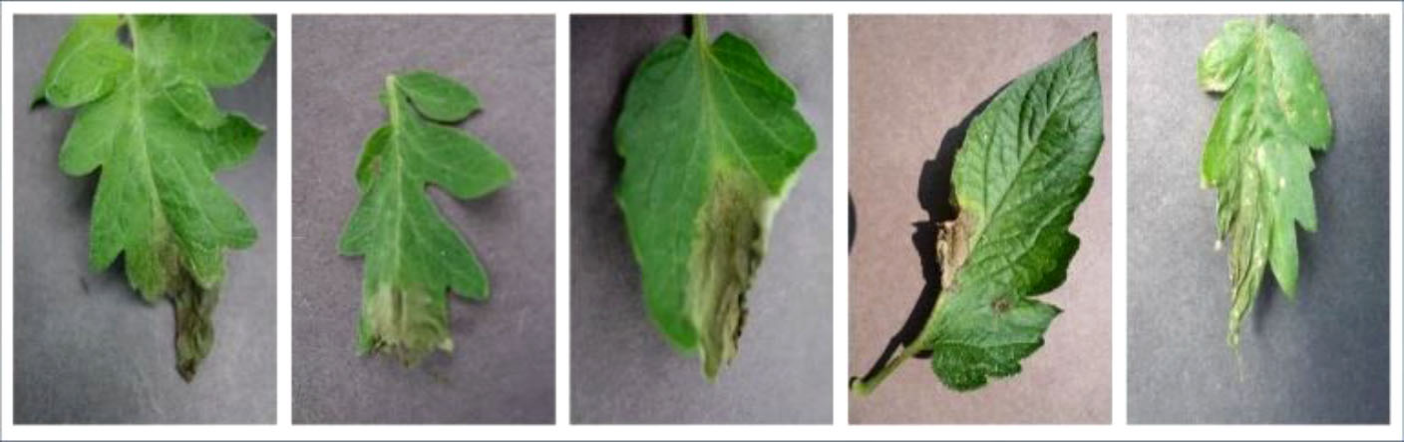
Some enhanced images using the AIM, NST, and GAN methods.

Additionally, the number of photographs in each category has been increased to 2500. In the PlantVillage dataset, the image of a leaf was collected in the positive direction. **Figure 1** displays illustrative enhancement images produced by AIM, NST, and GAN technologies.

**Figure 1**’s first two images are created and enhanced utilizing the AIM approach. **Figure 1**’s third and fourth images were created using DCGAN augmentation, while the final image was created utilizing NST technology. Using the hold-out cross-validation scheme, three sub-datasets of the plant village dataset are created for model training, validation, and testing purpose. **Table 2** displays the three sub-datasets details such as the number of images and the number of the image in a single class.

**Table 2:** Number of Images in the training, Validation, and Testing Set.

Dataset Name	Number of Images	Number of Images in Each Class
No. of Training Images	116,206	110250
No. of Validation Images	23241	1549
No. of Testing Images	22817	1521

In the following methodology section, the construction, explanation, and fine-tuning of a proposed DCNN model for disease identification in plant leafs utilizing hyperparameter fitting techniques and the PlantVillage dataset.

### 3.2 Proposed model

**Figure 2:**
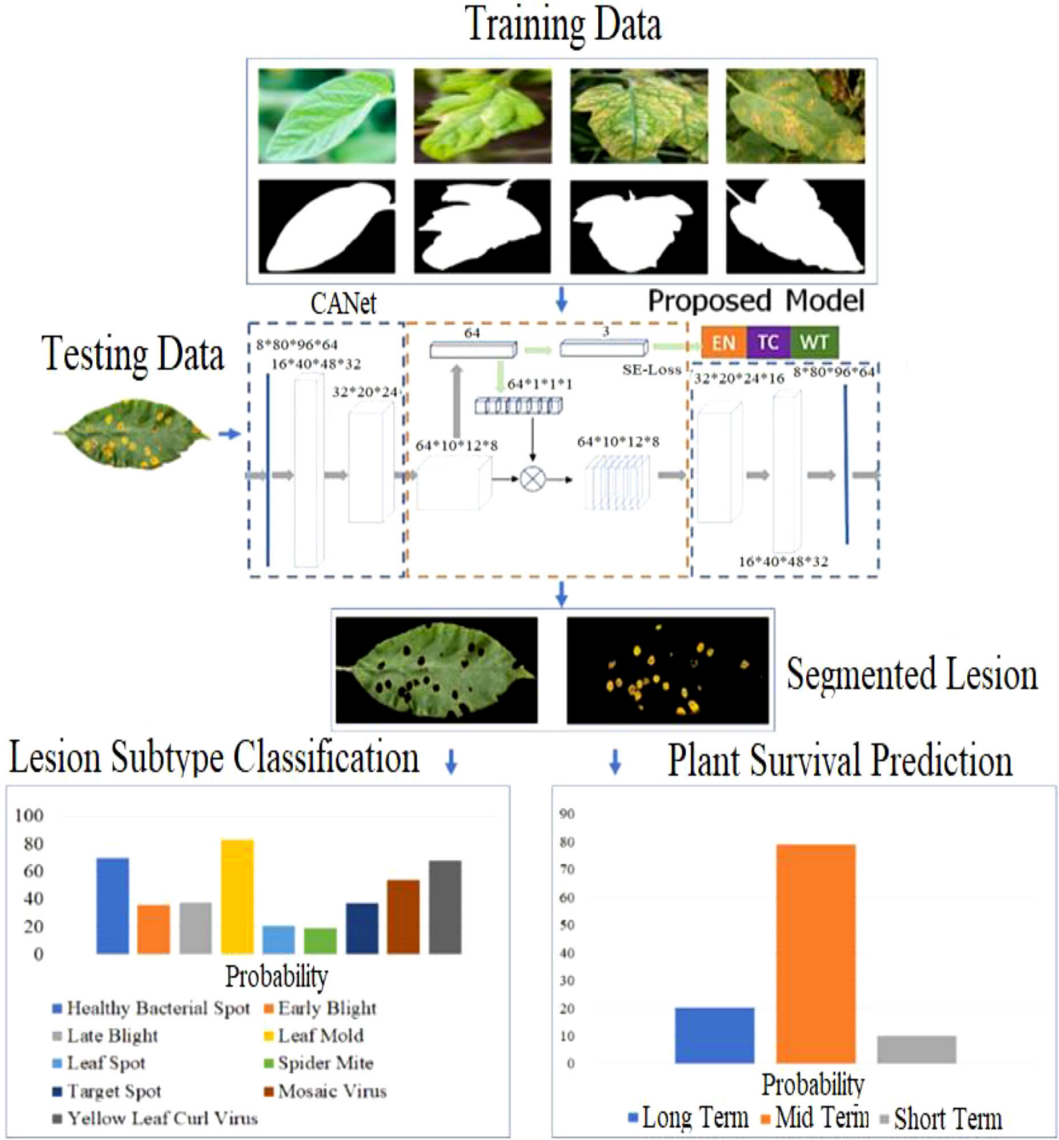
Overview of the methodology and overall workflow.

Numerous strategies for plant disease segmentation are described in the literature, including filtering-based, color-based, adaptive model-based, clustering, and regional convolutional neural network methods. Recently, approaches based on semantic segmentation have improved the segmentation of plant lesions. For plant lesion classification, structural and pathological pictures that are non-invasive are employed to classify plant leaf lesions. Predictions of overall survival assess the remaining lifespan of plants afflicted by prevalent illnesses. The majority of existing work relies on conventional regression models in machine learning such as Support Vector Machine and K-Nearest Neighbor. Our proposed framework can be summarized in **Figure 2** below. In the first stage, the training image and its essential facts are fed into the proposed training neural network; then, z-score normalization is performed exclusively on the lesion regions and differencing it from the min-max normalized image. CANet semantic segmentation model is used to detect the lesion area in a leaf image which is also depicted in **Figure 2**. Using segmented aberrant tissue, 3D CNN is utilized to classify leaf lesions. In conclusion, we employ the CANet front-end to extract high-dimensional data and then apply linear regression to make predictions about overall survival. Moreover, we assume that the model with the best performance in lesion segmentation would also attain higher accuracy in lesion subtype categorization and plant survival estimation, as the fact that CANet is utilized as a feature extractor in the segmentation and classification tasks, therefore we continue to use the same features with linear regression plant survival prediction.

### 3.3 Context-aware deep neural network

**Figure 3:**
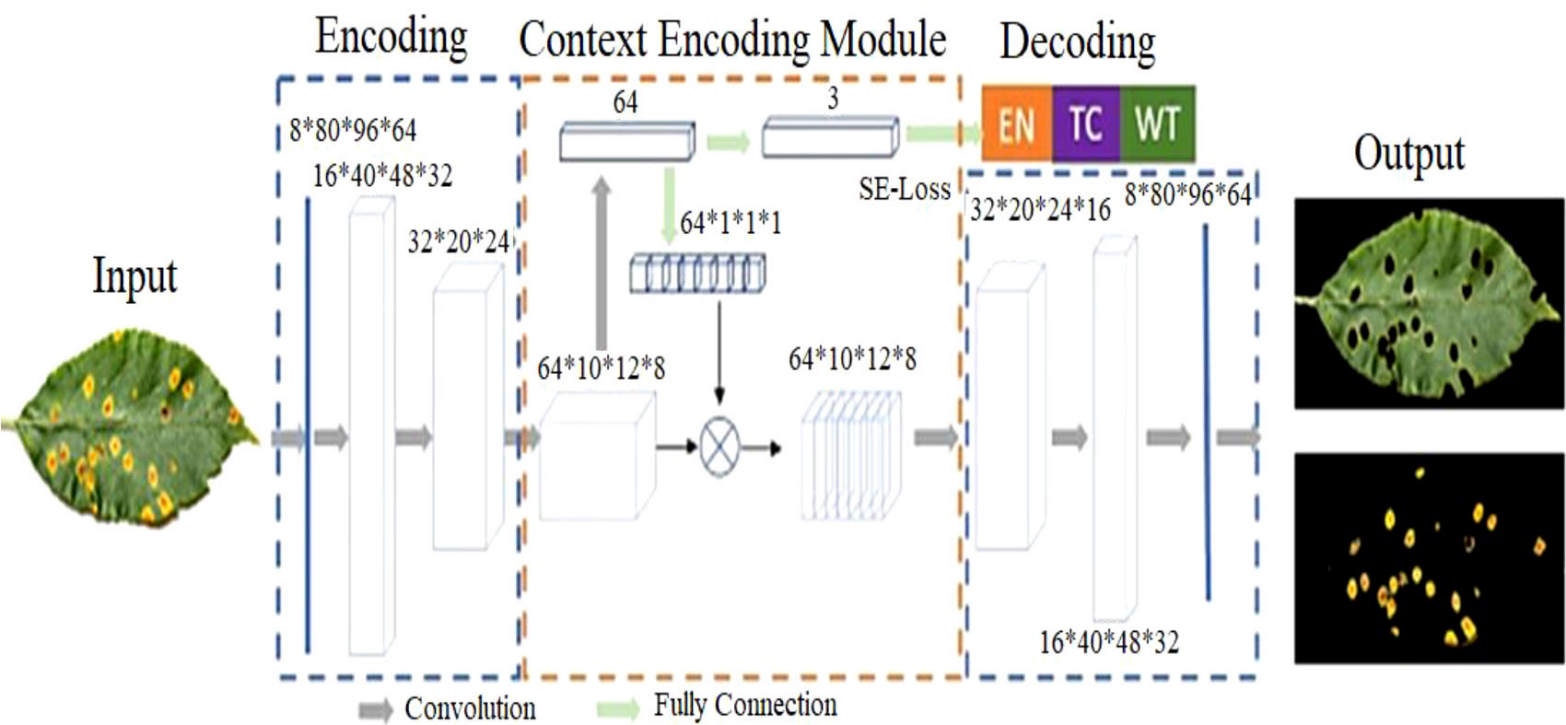
Proposed Leaf Lesion Segmentation model utilizing the CANet architecture.

This research provides an architecture for a context-aware convolutional neural network (CANet) that incorporates numerous image-processing tasks. Informed by contextual coding networks, the suggested architecture significantly improves plant lesion segmentation, subtype classification, and plant life survival prediction. **Figure 3** depicts the state-of-the-art CANet CNN with pertinent design parameters. The context coding module, which calculates the scale factor associated with representing all classes, is a crucial component of the proposed CANet. During training, these factors are simultaneously learned *via* the Lse-defined false regularisation loss. The scale factor captures global information for all classes and effectively learns to counteract potential training biases caused by unequal class representation in the image data.

Consequently, the ultimate features learning loss function module consist of two components:


(1) $$L=\;L_{dise}+L_{se}$$

Where L_dise_ is the DICE generated from the difference between the forecast and the underlying facts, and L_se_ represents the semantic loss. CANet is shared over the three pipelines, including plant leaf lesion segmentation, classification of plant lesion into healthy or ill, and lifespan estimation of plant, because of the intrinsic resemblance of each task and the likely overlay of valuable information. Consequently, the coding segment of the CANet CNN is utilized as a feature descriptor for plant survival estimation, whilst the plot holding the probabilities of plant lesion subregion created by the decoding segment is fed to the lesion subtype recognition model. The classification of lesion subtypes and the survival prediction pipelines use the CANet model with the best lesion segmentation performance.

### 3.4 CNN-based leaf lesion segmentation

**Figure 4:**
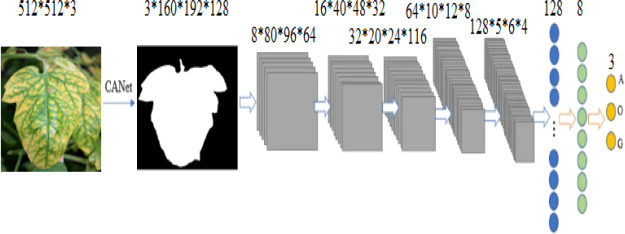
Overview of Semantic Segmentation for plant leaf lesion classification. In the first phase, the images are segmented by the CANet model which are then fed into the CNN classifier for disease subtype classification.

**Figure 4** depicts the context-aware deep learning algorithm proposed for leaf lesion segmentation. The suggested CANet captures global texture information and normalizes training failures with semantic loss. 19,36 The architecture comprises modules for encoding, contextual encoding, and decoding. From the input, the encoding module extracts high-dimensional characteristics. To standardize the paradigm, the context encoding module generates updated features and semantic losses. The decoding module reconstructs the entity map as a predicted output such that the difference between the predicted output and the input image can be computed as regularization. The proposed CANet offers an average DSC of 0.91 for ET, 0.90 for WT, and 0.95 for TC. **Table 3** show the plant leaf lesion semantic segmentation model.

**Table 3:** List of hyper parameters used for training plant leaf lesion semantic segmentation model.

Parameter	Value/Type
Optimizer	Adam
Initial Learning Rate	0.0001
Validation Data	Yes
Epochs	20
BatchSize	64
Shuffle Samples	Every Epoch

### 3.5 CNN-based leaf lesion classification

**Figure 4** depicts the foliar lesion classification procedure. Consequently, the CANet output is sent directly to a CNN-based classifier to classify lesion subtypes. Two fully connected layers follow five convolutional and clustering layers, and a classification layer with three outputs makes up the classification model. Except for the classification layer, which utilizes the softmax activation function, other layers employ ReLu activation. This study investigated several subtypes of foliar lesions, including bacterial plaque, early blight, leaf mold, target plaque, etc. During the testing phase, the suggested approach uses a DSC of 0.74. Using the recommended methodology, our test results placed second in the PlantVillage competition. **Table 4** show the CANet CNN model for plant leaf disease subtype classification.

**Table 4:** List of hyper parameters used for training CANet CNN model for plant leaf disease subtype classification.

Parameter	Value/Type
Optimizer	SGDM
Momentum	0.5
Initial Learning Rate	0.0001
Validation Data	Yes
Epochs	50
BatchSize	128
Shuffle Samples	True

### 3.6 A hybrid method for survival prediction

**Figure 5:**
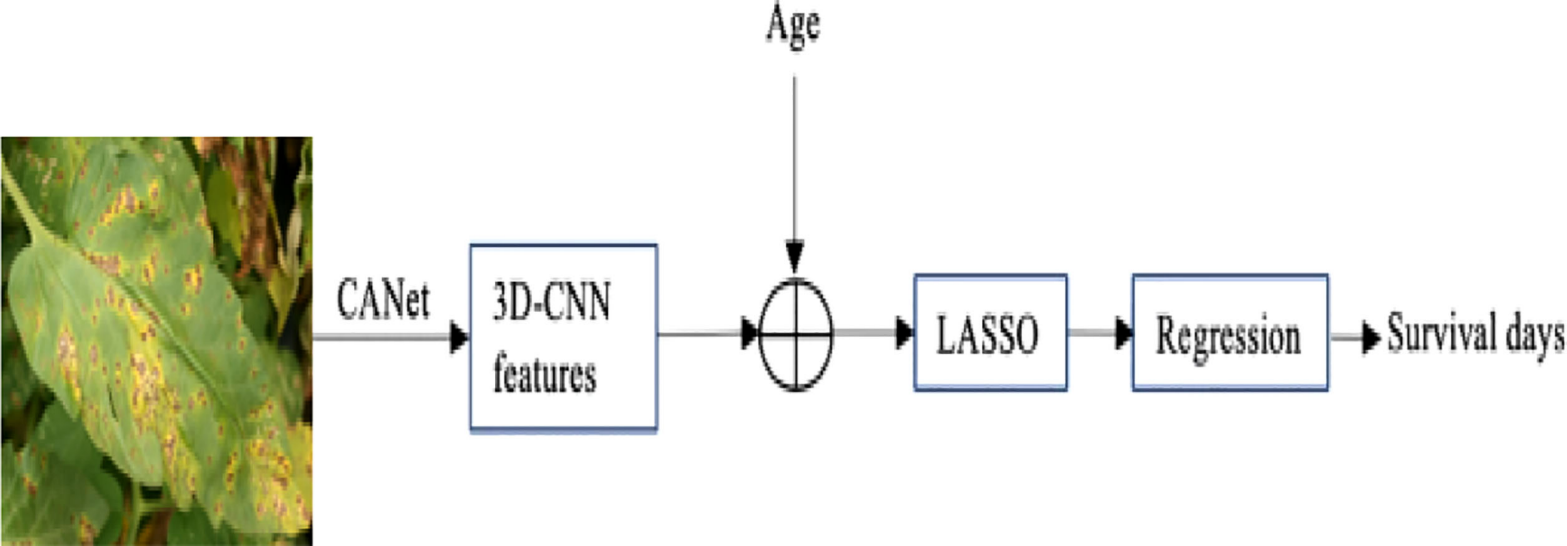
The overview of the proposed plant survival estimation mode is visualized. The CANet model extracts non-invariant features, also called 3D feature points. The plant age is added as additional information; the LASSO features selection method is used to select the optimal list of features from the feature vector. The final step is performed by a linear regression model that estimates the number of days for which the plant survives.

Instead of using typical machine learning approaches to extract features, we employ the suggested CANet to extract high-dimensional features. We believe that lesion segmentation characteristics correlate with overall survival. In addition to the CANet extraction feature, we leverage plant age as an additional feature. The LASSO approach is used to determine the number of days the plant will live by selecting more pertinent characteristics, it uses the features selection approach to select the most suitable features from the CNN features. The LASSO approach reduces the dimensions to features vector from 1x1000 to 1x241, which are fed in a regression model for training. Finally, we used linear regression to estimate overall survival based on the selected features, as shown in **Figure 5**. During the testing phase, the proposed technique demonstrated encouraging results with a Root Mean Square Error of 0.89. **Table 5**: shown the parameters linear regression model.

**Table 5:** List of parameters used for the training of linear regression model.

Parameter	Value
Attribute Selection Method	LASSO
Eliminate Colinear Attributes	Yes
BatchSize	50
Number Decimal Places	2
Ridge	1.0e-3

## 4 Experimental results

This section gives a comprehensive summary of the experiments’ outcomes to determine the proposed technique’s evaluation capabilities. This section also describes the dataset used to evaluate performance. The proposed framework is implemented in Python and runs on systems with Nvidia RTX 3090. CenterNet configuration on the PlantVillage dataset for classifying and scoring plant leaf diseases.

### 4.1 Evaluation metrics

When evaluating the effectiveness of the approaches we provide, we use a variety of evaluation metrics, including the Intersection Over Union (IoU), precision, accuracy, recall, and mean average precision (mAP). The accuracy of our proposed model is calculated as follows:


(2) $$Accuracy=\;\frac{TP+TN}{TP+FP+TN+FN}$$

Equation 7 is the mathematical equation for calculating the mAP score; the AP represents the average precision obtained by each class, whereas the s represents the test image and S is the number of total test images.


(3) $$mAP=\;\sum_{i=1}^{T}AP\left(T_{i}\right)/T$$

The equation below represents the Inter over Union ratio.


(4) $$Precision=\;\frac{TP}{TP+FP}$$

(5) $$Recall=\;\frac{TP}{TP+FN}$$

(6) $$IoU=2*\frac{TP}{FN+FP+TP}$$

### 4.2 Performance evaluation of plant disease localization

**Figure 6:**
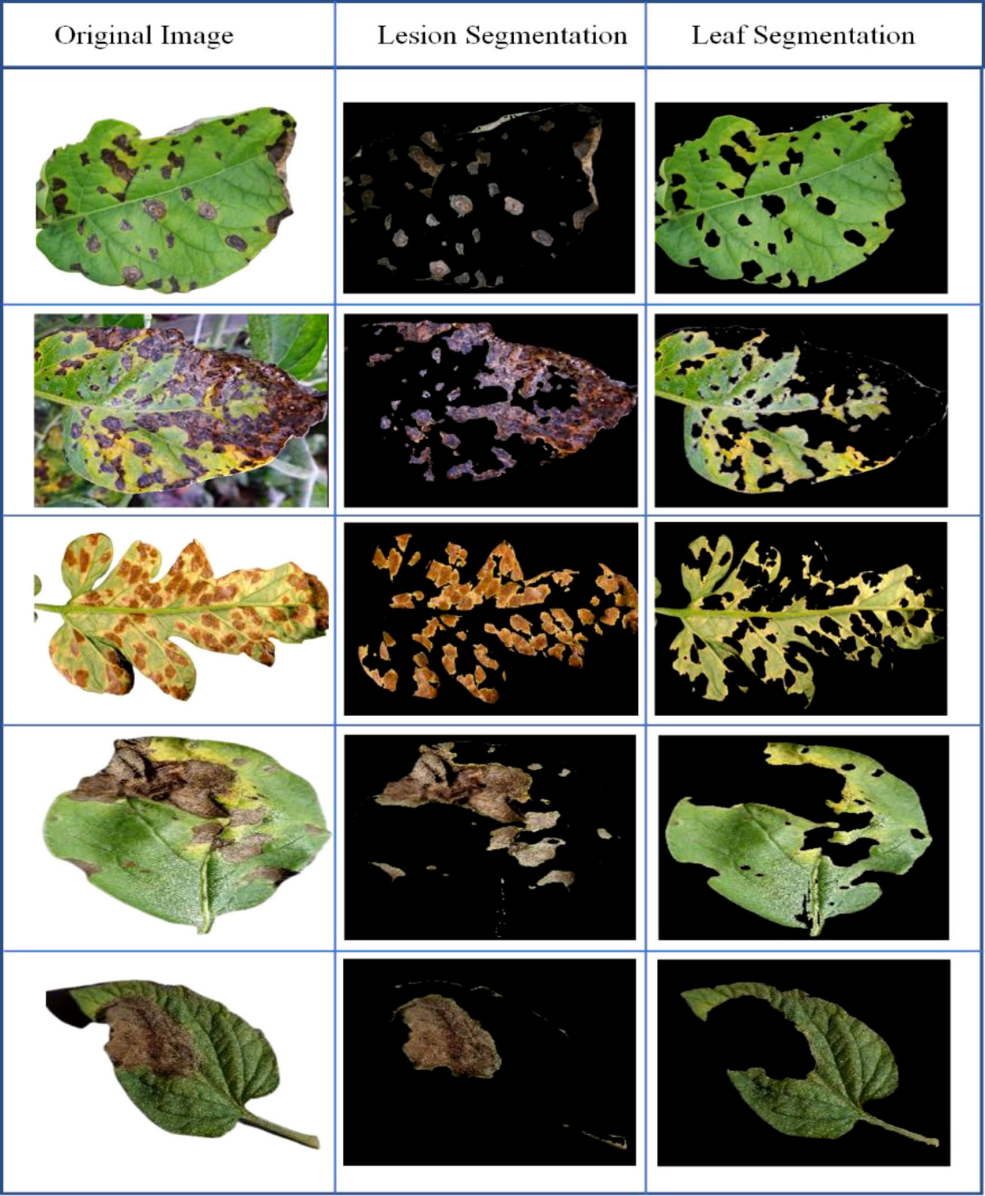
Proposed model segmentation results.

**Figure 7:**
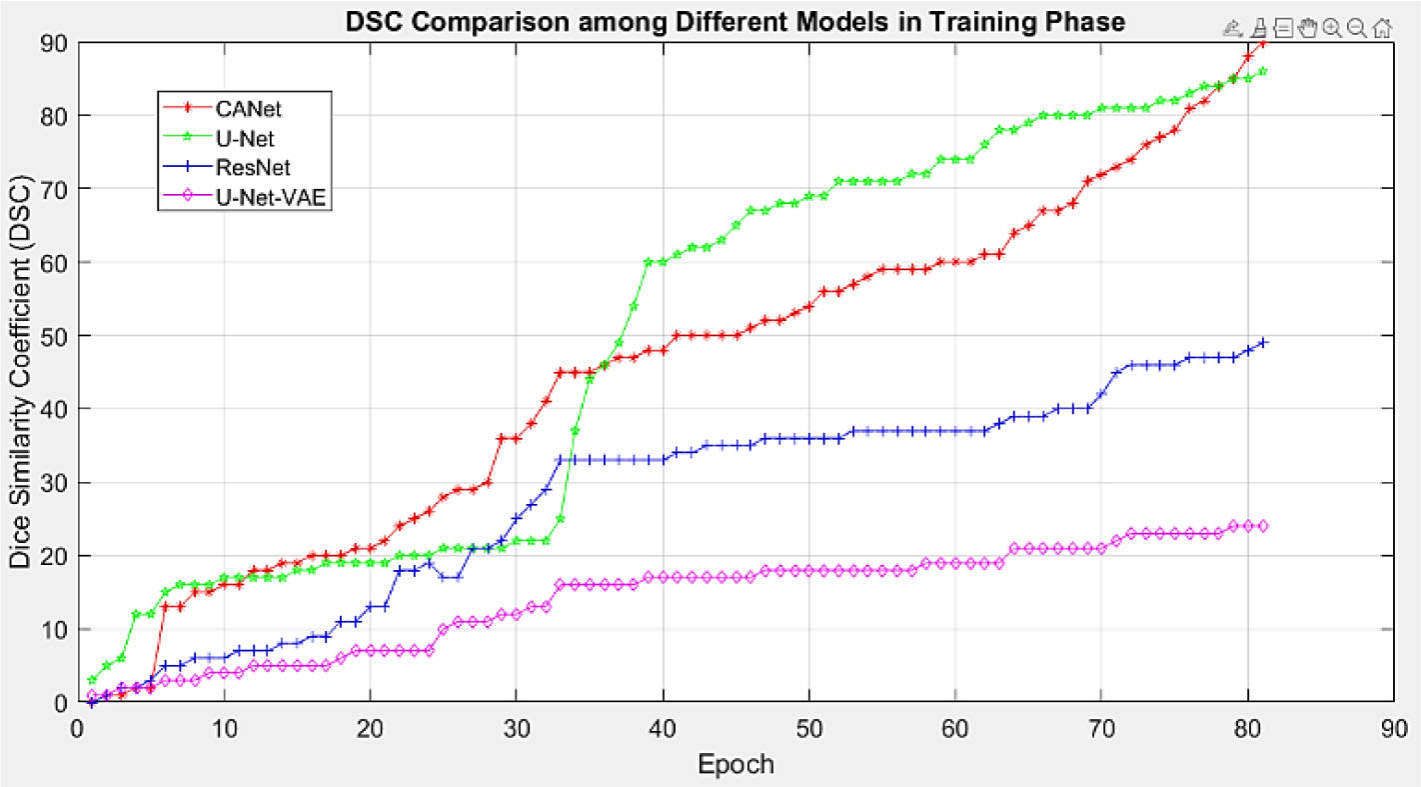
Proposed CANet CNN Model performance comparison using the DICE coefficient metric.

Establishing an efficient model for the automatic identification of agricultural illnesses depends heavily on accurately detecting different plant diseases. For this purpose, we experimented to determine the suggested technology’s placement capacity. All samples from the PlantVillage dataset were evaluated, and the samples are displayed in **Figure 6**. The given results demonstrate that Custom CenterNet can accurately detect and identify many types of plant illnesses as shown in **Figure 7**. In addition, the suggested method is resistant to numerous post-processing attacks, including blur, noise, light and color shifts, and image distortion. CenterNet’s positioning capabilities enable accurate identification and localization of various plant diseases. We employ mAP and IOU indicators to quantify the positioning capabilities of the proposed technology: mAP and IOU. These indicators aid in analyzing the system’s performance in diagnosing various plant diseases. Specifically, we acquire mAP and IOUs of 0.99 and 0.993%, respectively. The visual and numerical results indicate that the technique can reliably detect and classify plant illnesses. The performance analysis of the proposed leaf lesion segmentation is shown in **Table 6**.

**Table 6:** Performance Analysis of the proposed leaf lesion segmentation model.

Performance Metric	Results
Accuracy	92%
Precision	95%
Recall	91%
IoU	90%

### 4.3 Plant disease classification results

To detect pepper plant disease, a binary classification CNN model is trained. Due to the small dataset size (Fewer Classes), the model was efficiently trained for classifying healthy and unhealthy pepper plants through leaf images. The pepper plant disease detection model confusion matrix is shown in **Table 7**. The model achieved higher accuracy in the detection of bacterial spot disease. In comparison, for the detection of the healthy class, the proposed achieved higher accuracy and f-measure than the unhealthy class. The detailed performance analysis can be seen in **Table 8** which consists of classwise and average values of four performance metrics.

**Table 7:** Proposed model confusion matrix for Peppers plant disease classification.

	Healthy	Bacterial Spot
**Healthy**	2587	143
**Bacterial Spot**	190	2312

**Table 8:** Detail Performance by class for pepper disease detection.

	Accuracy	Precision	Recall	F-Measure
Healthy	93.63%	94.76%	93.15	93.95315
Bacterial Spot	88.59%	92.40%	94.17	93.28223
Average	91.11%	93.58%	93.66%	93.61

**Figure 8:**
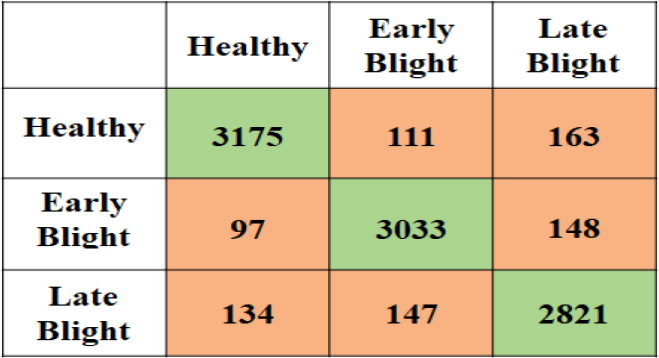
Proposed model confusion matrix for Potato plant disease classification.

The second experiment is performed on the potato leaf image to classify them into healthy or Early Blight or Late Blight Classes. **Figure 8** is the confusion matrix created using the actual plant condition and model predicted values for each individual class the True Positive Rate (TP), False Positive Rate (FP), False Negative Rate (FN), and True Negative rate (TN) are calculated. Using these measures for the proposed model, the accuracy, precision, recall, and F-measure are calculated, which can be seen in **Table 9**. Due to the data imbalance, the model’s performance is assessed using the F-Measure. The F-measure finally evaluates the proposed model’s correct detection rate, indicating that the proposed model slightly performed better in detecting healthy plants than the other unhealthy class (Early Blight, Late Blight).

**Table 9:** Detail Performance by class for potato disease detection.

	Accuracy	Precision	Recall	F-Measure
Healthy	94.86214	92.05567	93.21785	92.63311
Early Blight	92.59859	92.52593	92.16044	92.34282
Late Blight	91.55251	90.94133	90.07024	90.50369
Average	93.01	91.84	91.81	91.82

**Figure 9:**
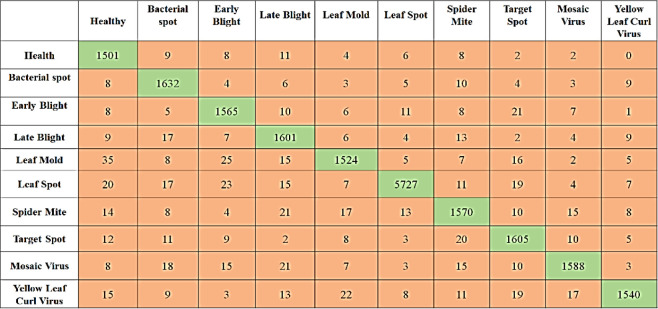
Proposed model confusion matrix for Tomato plant disease classification.

**Table 10** presents a quantitative evaluation of the efficacy of the proposed model in identifying diseases that can affect tomato plants. Images of tomato plant leaves in both healthy and unhealthy states (bacterial spot, Target spot, mosaic virus, etc) are used in the experiment. In order to evaluate the effectiveness of the model, we first compute the four standard performance evaluation metrics using the confusion matrix values presented in **Figure 9**. Because of the imbalance in the data, the performance of the model is evaluated using the F-Measure. The proposed strategy was successful in achieving higher detection accuracy as well as the f-measure for both the Healthy and Unhealthy classes.

**Table 10:** Detail Performance by class for Tomato disease detection.

	Accuracy	Precision	Recall	F-Measure
Healthy	99.13739	96.77627	92.08589	94.37284
Bacterial Spot	99.19452	96.91211	94.11765	95.49444
Early Blight	99.08788	95.3106	94.10704	94.70499
Late Blight	99.03394	95.75359	93.35277	94.53794
Leaf Mold	98.9702	92.81364	95.01247	93.90018
Leaf Spot	98.79526	97.89744	98.99741	98.44435
Spider Mite	98.88953	93.45238	93.8434	93.64748
Target Spot	99.04419	95.25223	93.96956	94.60654
Mosaic Virus	99.14418	94.07583	96.12591	95.08982
Yellow Leaf Curl Virus	99.14632	92.93905	97.03844	94.94451
Average	99.04	95.18	94.86	94.97

### 4.4 Performance of plant survival prediction

The plant survival estimation is performed using a linear regression model. The survival is estimated using the model trained on the entire dataset consisting of 1000 attributes which are the features extracted using the CANet CNN model. The plant survival is also estimated using a regression model followed by the LASSO features selection method that reduces the feature vector size and tries to select the optimal attributes. The feature vector dimensions by the LASSO method are reduced to 1x241, shown in **Table 11**.

**Table 11:** Performance analysis and comparison of survival estimation model with LASSO and without LASSO approach.

Metric	Linear Regression	Linear Regression + LASSO
Correlation Coefficient	2.04	0.91
Mean Absolute Error	1.54	0.65
Root Mean Squre Error	2.97	0.89

## 5 Discussion

Deep learning-based algorithms have attained state-of-the-art performance in numerous sectors where they have been widely implemented. However, leaf lesion segmentation provides numerous specific challenges:

◼Image quality has a significant effect on segmentation efficiency. For instance, blurry visuals result in negative effects.
◼Image preprocessing steps have an effect on performance as well. For instance, standardization of intensity across cases is crucial for lesion segmentation.
◼The heterogeneity of lesion tissue may provide a formidable obstacle to the development of an efficient approach.
◼Unbalanced data is a common complication for the use of deep learning.
◼**Figure 2** depicts the data distribution from our studies during the training phase for lesion categorization and overall survival prediction. Cases of Healthy account for more than fifty percent of the training data. In survival prediction, the range of mid-term survival days is insufficiently broad relative to the short- and long-term ranges, resulting in an imbalance of data. This data disparity may lead to misclassification. In the segmentation process, lesion samples are typically substantially larger than those of other defective tissues. To solve the potential data imbalance issue in lesion segmentation, we implement plant leaf lesion segmentation based on leaf subregions as opposed to employing each defective tissue separately.

**Figure 10:**
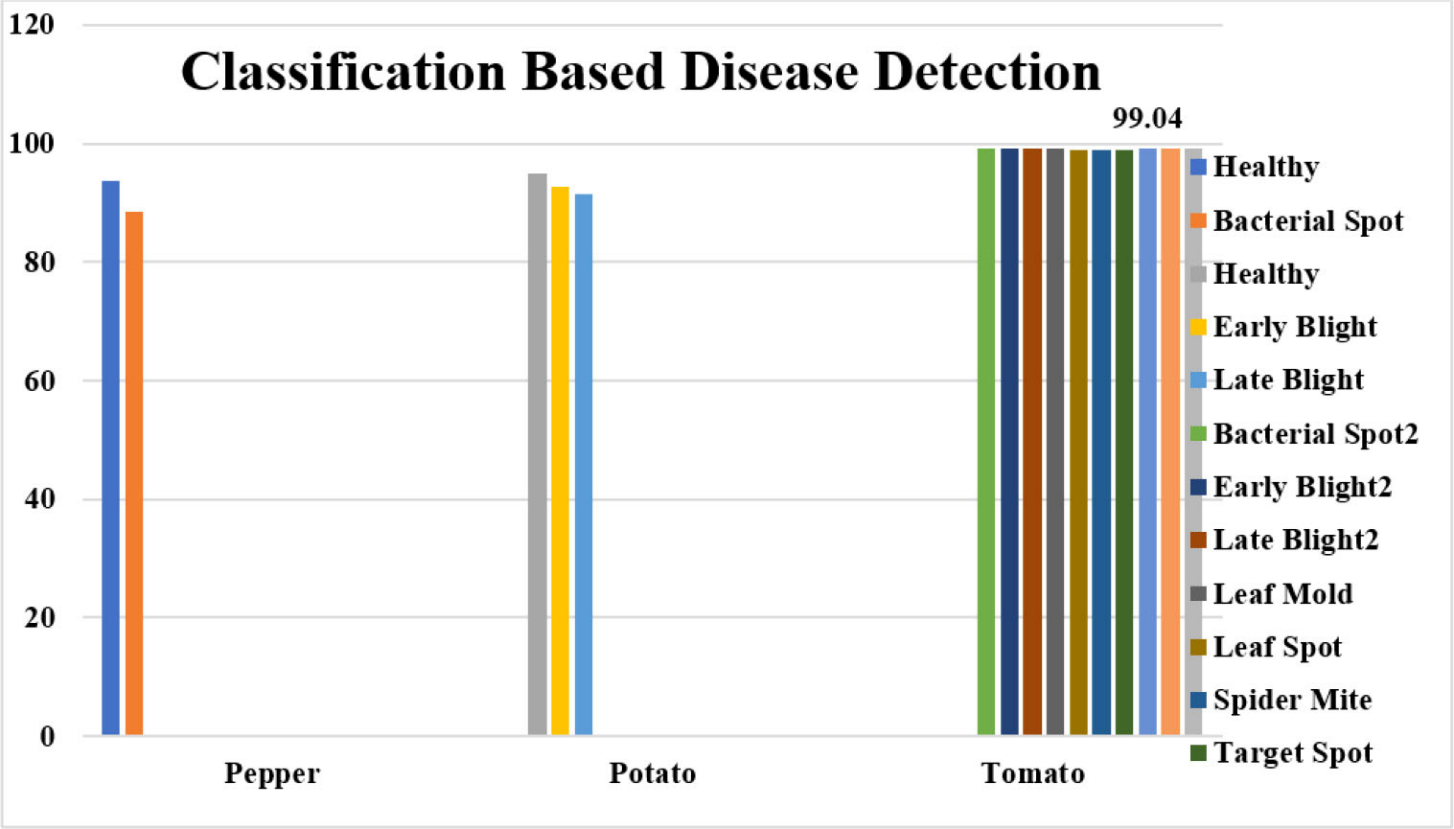
Performance Comparison of the proposed deep learning model for pepper, potato, and tomato plant disease detection.

The fundamental challenge with disease classification is the lack of data. Even after increasing the training sample size using data augmentation approaches, 110250 examples may not be sufficient for deep learning in this work. Similar data deficiency issues exist for global survival prediction. In the PlantVillage Challenge training phase, only ten classes are accessible. In addition to the deep learning-based approach, we implement global survival prediction using a conventional machine learning method by extracting features such as gray-level co-occurrence matrix (GLCM), intensity, etc., applying LASSO to select features and then using linear regression for survival prediction. We compare the outcome to our proposed method’s outcome. The comparison demonstrates that the performance of the proposed strategy is superior. In this paper, we also assess the influence of various diseases on overall survival. There are three classification models trained for the classes of peppers, potatoes, and tomatoes. The detection accuracy of models for pepper, potato, and tomato plants is 99.11%, 94.01%, and 99.04%, respectively shown in **Table 12**. The proposed deep-learning model for pepper, potato, and tomato plant disease detection is shown in **Figure 10**.

**Table 12:** Performance Comparison of the proposed disease subtype classification model with some state-of-the-art models.

Study	Model	Accuracy
(Wu et al., 2020)	DCGAN+CNN	94.33%
(Sibiya and Sumbwanyambe, 2021)	N-Fuzzy+CNN	89%
(Islam et al., 2022)	Parallel CNN	98%
(Brahimi et al., 2017)	DNN	99.18
Proposed CANet		99.04%

## 5 Conclusion

This study investigates several plant disease diagnostic and analysis tasks using deep learning and plant leaf imagery. These tasks involve segmenting the leaf lesion area, classifying the lesion into its subtypes, and predicting the plant’s overall survival. We built a context-aware 3D CNN that extracts and classifies high-dimensional, non-invariant characteristics from a plant leaf image to identify the disease type. Similarly, a unique method is established utilizing the regression model to predict long-term, short-term, and intermediate-term plant survival. The features learning block of the CANet CNN model extracts features, reduces the dimension of the features vector by picking only the optimum features, and employs the LASSO features selection algorithm. The PlantVillage Dataset comprises numerous photos of crop leaf diseases. This study has validated the suggested model using three different plant diseases: pepper, potato, and tomato. The pepper plant has only two classes, but the potato and tomato plants have multiple classes. The suggested model achieves a DICE coefficient of 90% while segmenting plant leaf lesions. The classification accuracy for detecting pepper illness is 91.11%, for detecting potato disease is 93.01%, and for detecting tomato, the disease is 99.04%. Consequently, the improved accuracy suggests that the suggested method applies to the PlantVillage dataset and other datasets for lesion segmentation, classification, and plant survival calculation.

## Data availability statement

The datasets presented in this study can be found in online repositories. The names of the repository/repositories and accession number(s) can be found in the article/supplementary material.

## Author contributions

MS, BS, TH, AU, AA, IS, TG, and FA conceptualized this study, conducted experiments, wrote the original draft, revised the manuscript, performed the data analysis, supervised the work, and revised the manuscript. FarA designed the experimental plan, supervised the work, and revised the manuscript. All authors have read and agreed to the published version of the manuscript.
